# Approach to the child with fatigue: A focus for the general pediatrician

**DOI:** 10.3389/fped.2022.1044170

**Published:** 2022-12-02

**Authors:** Laura De Nardi, Maria Andrea Lanzetta, Elena Ghirigato, Egidio Barbi, Giulia Gortani

**Affiliations:** ^1^ University of Trieste, Clinical Department of Medical Surgical and Health Science, Trieste, Italy; ^2^ Department of Pediatrics, Institute for Maternal and Child Health IRCCS “Burlo Garofolo”, Trieste, Italy

**Keywords:** fatigue, tiredness, fatigability, weakness, tired child, weariness, drowsiness

## Abstract

**Background:**

Fatigue is a common, nonspecific complaint commonly used to describe various conditions, ranging from a vague, subjective sense of weariness to muscular weakness, fatigability, exercise intolerance or excessive daytime somnolence. Despite its high frequency in the general population, literature addressing the approach to the child with fatigue from a general pediatrician perspective is poor. We herein propose a review of the available evidence on the topic, providing a practical framework to assist physicians in dealing with the issue.

**Methods:**

Data were identified by searches of MEDLINE, UpToDate, Google Scholar and references from relevant articles. Articles published between 1990 and 2021 were considered, prioritizing systematic reviews and meta-analyses. Then, an empirically-based model of approaching the tired child was proposed according to our center experience.

**Results:**

To correctly characterize the meaning of fatigue reporting, specific clues from history and physical examination should be emphasized. Duration, severity, and the age at onset are to be considered. Then, specific queries about everyday activities, sleep hygiene and social domain could be useful in reaching a specific diagnosis and offering an appropriate treatment.

**Conclusions:**

We suggest a pragmatic approach to fatigue in children based on age assessment, targeted questions, physical examination clues, and some laboratory first-level tests. This could provide pediatricians with a useful tool to discriminate the broad etiology of such a complaint, disentangling between psychological and organic causes. Further studies are needed to investigate the predictive value, specificity and sensitivity of this diagnostic workflow in managing the child with fatigue.

## Introduction

Tiredness, weariness, weakness, fatigability and fatigue are words commonly used in the everyday vocabulary, sometimes mistakenly as synonyms, to describe a sense of difficulty or inability to initiate, maintain or complete tasks which require mental and/or physical energy. However, such different meanings should not be interchangeable to the physician. For example, the reduced capacity to maintain an activity, defined as easy fatigability and evocative of a myasthenic syndrome, should be distinguished from the inability to initiate or perform a specific action, which may underlie a neurological or muscular disease. Also, discriminating between psychological and physical tiredness can be challenging, especially among adolescent patients. Furthermore, fatigue can be secondary to an almost unlimited range of acute and chronic diseases encompassing cardiological, pulmonary, endocrinological, gastrointestinal, nephrological, rheumatological and metabolic conditions, cancer, and psychological/psychiatric comorbidities (1–7).

Due to the broad etiology and subjectivity of its reports, the epidemiology of fatigue is also rather challenging to define. A generic complaint of fatigue represents about 10%–15% of primary care consultations among adults (8). Notably, in adults, chronic fatigue is strongly associated with psychiatric disorders, with two-thirds of people suffering from long-lasting fatigue being also affected by psychiatric comorbidities (9). The prevalence of children complaining of fatigue falls to about 4.4%, depending on sex and age, with the highest frequency amongst adolescent females (10, 11). Remarkably, the reported number of pediatric patients complaining of psychological fragility has continuously been increasing in the last decades (12–14). Recent studies found fatigue to be more prevalent in children affected by chronic diseases, reaching a rate of 21.2% in those with cystic fibrosis, autoimmune diseases and post-cancer treatment, and 25% in pediatric rheumatic disease (15, 16). The available literature has mainly focused on quantifying and managing fatigue in the broader context of childhood chronic diseases (3–6, 17), while less is written on the definition and categorization of fatigue complaint *per se*. Another significant proportion of studies deals with chronic fatigue syndrome (CFS), a blurred defined and debated clinical entity, with specific diagnostic criteria and significant overlap with psychiatric and functional disorders (18, 19).

Due to the inherent subjectivity of the symptom, quantification and assessment of fatigue is even more challenging in the pediatric population for the possible ambivalence between patient's and parental reports (20). Physicians, in their daily practice, use age criteria, history and physical examination clues to discriminate the different hypotheses and establish if and which tests are to be performed. However, despite the high frequency of such a complaint, we are not aware of any study or review suggesting a general pediatric guideline in this perspective. Applying a diagnostic algorithm to the symptom of fatigue could help to discriminate its multiple causes, possibly leading, on the one hand, to an early diagnosis of specific organic conditions and, on the other, to the timely unmasking of possible mental health problems. The latter is especially relevant, as symptom persistence is reported to lead to prolonged school absence, isolation and loss of activities resulting in the risk of academic failure and development of psychiatric conditions in adult age (21–23). This work aims to review the available evidence on the topic and provide a pragmatic framework to assist the pediatrician in dealing with the child complaining of fatigue as the main isolated symptom, both in primary care setting and in the emergency department. Further studies will be needed to assess the effectiveness of this approach and improve it.

## Materials and methods

Data were identified by searches of MEDLINE, UpToDate, Google Scholar and references from relevant articles. The following search string was used for MEDLINE: (“fatigue” OR “weakness” OR “tiredness” OR “weariness” OR “drowsiness”) AND (“child” OR “pediatric” OR “paediatric” OR “children” OR “kid” OR “toddler”). We also combined the keywords “tired child”, “child with fatigue”, “paediatric fatigue”, “pediatric fatigability”, (“chronic fatigue” AND “children”), (“medication” OR “drugs” AND “fatigue” AND “children”), (“sleep disturbances” AND “children”) for the all databases.

Over 2000 articles were initially found through this search strategy, which included articles published between 1990 and 2021 in English and other languages, provided that an English abstract was available. With the aim of providing a practical decision-making strategy, we focused on articles dealing with fatigue as the main and isolated generic complaint, excluding papers related to fatigue in the context of already known chronic diseases, and fatigue related to Coronavirus disease (COVID-19). We prioritized systematic reviews and meta-analyses, and peer-reviewed literature in the public domain. We also included clinical and narrative review articles to provide more details on the topic to the readers. Only two not English articles were included and translated by native speakers. The examination of the literature was mostly focused on the pediatric population. However, when considering broader concepts applicable to both pediatric and adult patients, such as pathophysiology of fatigue and medication side-effects, the search was not restricted solely to pediatric literature. After the exclusion of duplicates, case reports and small case series, we restricted the results to 77 papers. Search strategy and literature results are synthetized in Table S1 and S2 (see Supplementary file S1).

Then, an empirically-based model of approaching the tired child was proposed according to our center experience and optimized with input from literature evidences. Since our literature search did not retrieve any previous attempt to establish a similar decision-making tool, we drew some information about fatigue assessment and useful lab tests from Guidelines on Chronic Fatigue Syndrome (24–27). Indeed, the CSF diagnosis requires the exclusion of all the other possible causes of fatigue, and some anamnestic elements, red flags and lab tests reported in our algorithm are the same included in the CSF diagnostic workout, which have a level of evidence D according to Delphi consensus (24). Besides that, other two works we considered very useful in the creation of such a perspective paper were drawn from UpToDate (28,29). We then integrated this information with our pediatric experience.

## Classification and pathophysiology of fatigue

Fatigue may be physiological or pathological. Brief periods of fatigue, occurring in approximately 15%–25% of the general population, can be secondary to situational stressors with an identifiable cause, such as physical exercise, acute febrile or flu-like illness, mental stress, or sleep deprivation (30, 31). In contrast to physiological fatigue, which is self-limited and rapidly resolved by treating the underlying factors, pathologic fatigue can be prolonged (lasting one to five months) or chronic (lasting six months or longer). Perception and performance are the two primary domains that contribute to fatigue classification. Indeed, it can be mental (due to a subjective sense of perceived fatigue) or physical (as an objective and measurable phenomenon) (32). Homeostatic and psychological factors can influence mental fatigue. Potential homeostatic contributors to the perception of fatigue include energetic, inflammatory, and neural feedback such as glycogen depletion, increased brain temperature, accumulation of ammonia, inflammatory cytokines, increases in serotonin, and decrements in dopamine among hypothalamic networks. Psychological factors include attitude, motivational influences, endurance, resilience, expectations, concentration, intention and mood. These aspects can be affected by psychiatric diseases such as depression, psychosis, addiction or burnout syndrome manifesting with a fatigue complaint (32–34). Physical fatigue, on the other hand, can be secondary to neurological or non-neurological disorders. When considering neurological causes of fatigue, they can be due to central or peripheral mechanisms (35). Central neurological fatigue depends on a progressive decline in voluntary activation of muscles; it may result from dysfunction of specific cortical or subcortical networks and may be due to spinal or supraspinal disfunction (31, 33, 34). The mechanism at the base of central fatigue disorders may depend on increased inhibitory interneuron input to the motor cortex, delayed central conduction from a demyelination process, poor coordination of motor unit firing and increased negative feedback from muscle afferent sensory neurons, or impaired recruitment of motor units (33). Examples of diseases characterized by central fatigue are multiple sclerosis, cerebrovascular ischemia, mitochondrial disorders, hereditary spastic paraplegias, spinocerebellar ataxias, central nervous system (CNS) infections and tumors, and traumatic brain injury. Central neurological fatigue can sometimes overlap with the previously mentioned mental fatigue. A motivational input actually activates a facilitation system to increase motor output from the primary motor cortex to overcome supraspinal fatigue and the sensory inhibition system from the peripheral system.

Peripheral fatigue is usually secondary to second motor neuron dysfunction, peripheral nerves, neuromuscular junction, or muscular diseases. The responsible mechanisms include axonal loss, demyelination process, or conduction block. In contrast, muscular mechanisms include loss of electrical conduction along the muscle membrane to the T-tubule system, impaired calcium release from the sarcoplasmic reticulum, altered interaction between actin and myosin, reduced calcium reuptake, and altered oxidative phosphorylation or glycolysis processes (33). Diseases that may present with peripheral neurological fatigue include second motor neuron dysfunctions, polyradiculopathies, plexopathies, polyneuropathies, neuromuscular transmission diseases, myopathies, or rhabdomyolysis (31, 36). Muscle weakness may be secondary to accumulation of intracellular lactate and hydrogen ions with consequent acidosis, depletion of glycogen, ATP, phosphocreatine, calcium conductance changes, and reactive oxidative species (ROS) (31).

These mechanisms are, for example, involved in glycogen storage disorders or mitochondrial myopathies. Finally, neurological fatigue may subtend at least two distinct subtypes that can coexist and should be sought: easy fatigability and true muscle weakness. The former is defined by the incapability to maintain muscle strength for prolonged periods of time and is typical of neuromuscular junction disease (i.e., myasthenia gravis). The latter, presenting as a partial or complete loss of muscle strength needed to start an activity, may be associated with muscle hypertonia and hyperreflexia in first motoneuron diseases, or with muscle hypotonia, hypotrophy, hyporeflexia with or without fasciculations in second motor neuron diseases and myopathies (see Table S3, Supplementary file S2).

Non-neurological diseases, instead, can cause fatigue through some of the mechanisms previously reported as pathogenetic of mental fatigue: pro-inflammatory cytokines, anemia, hormone deficiencies, poor nutrition, vitamin deficiencies, toxins, and poor tissue perfusion (37). These phenomena may be triggered by infectious, hematologic, gastroenterological (celiac disease, Chron disease), rheumatological, and endocrine disorders (diabetes, Addison's disease, hypothyroidism, hypopituitarism), as well as malignancies, and heart or kidney failure. In these cases, fatigue is rarely an isolated complaint, and the presence of one or more other symptoms will help with the diagnosis. One further possible cause is drug-related fatigue (38). For some medications, such as benzodiazepines, antiepileptics, neuroleptic and selected anti-hypertensives, drug-related fatigue is an on-target side effect (39–41). When considering old generation anti-histamines or non-selective beta-blockers instead, fatigue and sedation are off-target side effects due to, in the former case, to binding of H1 receptors in the CNS, and in the latter case, to alpha-blockade activity (41, 42). Amongst benzodiazepines, fatigue has been described (15%–30%) with clonazepam, clobazam, lorazepam and diazepam. When considering anti-epileptics, fatigue is most common with phenobarbital, primidone, vigabatrin, gabapentin and levetiracetam (40). Amongst anti-hypertensives, instead, sedation has been described as an adverse effect of clonidine and labetalol (43). Sedation is quite common also with second-generation antihistamine drugs, especially cetirizine, which is widely used in the pediatric population (44).

## The diagnostic workflow

**Figure 1:**
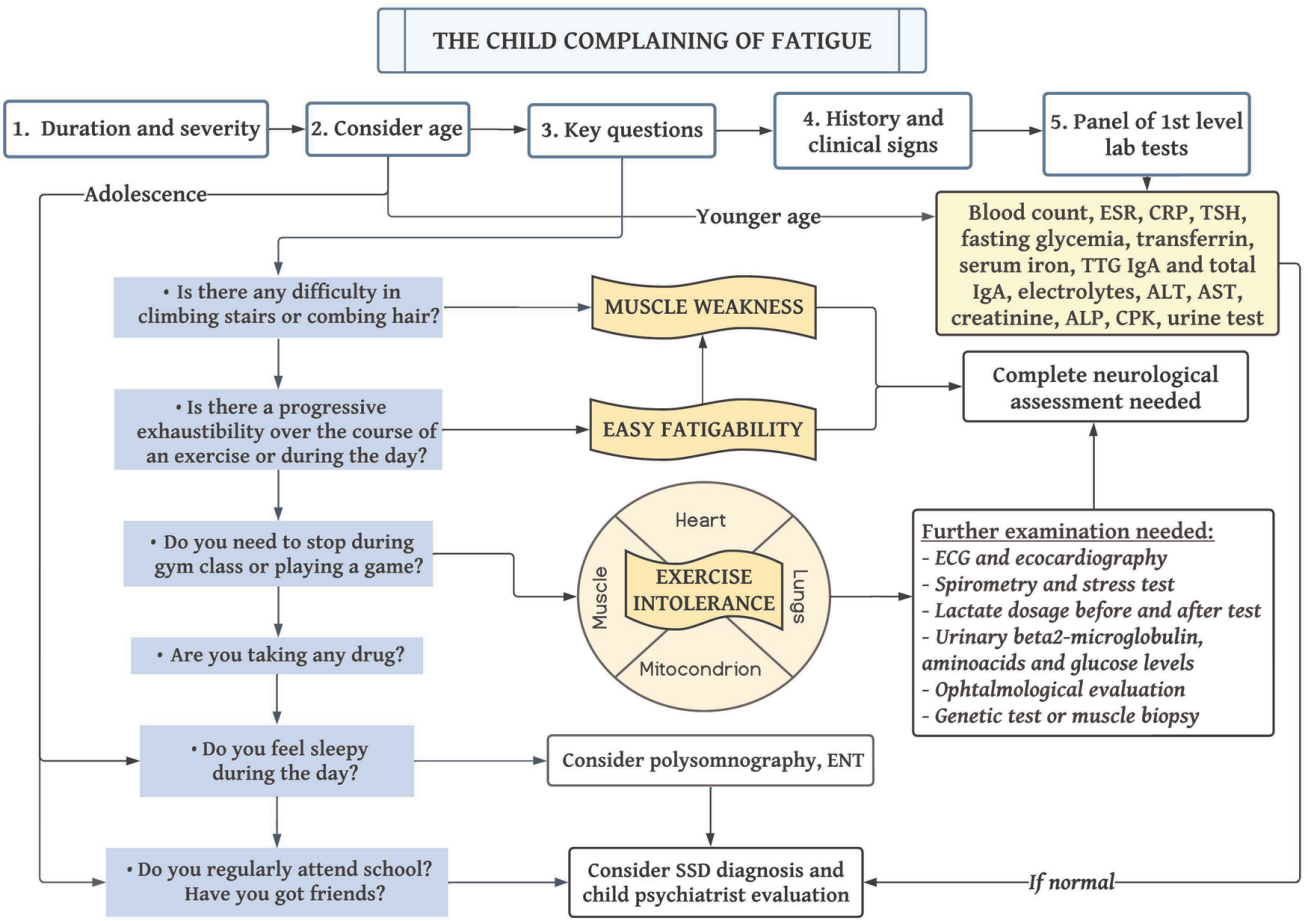
**Proposal of a diagnostic flowchart to assess the child complaining of fatigue**. Abbreviations: ESR, erythrocyte sedimentation rate; CRP, C-reactive protein; TSH, thyroid-stimulating hormone; TTG, tissue transglutaminase; AST, aspartate transaminase; ALT, alanine aminotransferase; ALP, alkaline phosphatase; CPK, creatine phosphokinase; ECG, electrocardiogram; ENT, ear nose and throat; SSD, somatic symptom disorder.

Figure 1 summarizes a proposal of a decision-making algorithm.

### Consider fatigue duration and severity

An isolated unspecific sensation of mild to moderate fatigue lasting less than one month, without any history or physical examination red flags, should be deferred for further evaluation at a later follow-up, as in the majority of the cases, it is self-resolving (45). It is the most common complaint to the general pediatrician and may be related to subclinical viral infection or temporary alterations in habits and environmental conditions, such as stress due to school or other extra-curricular activities.

### Age matters

Age is a crucial element to consider. Adolescence *per se* represents a delicate and complex moment in life, marked by increased self-identity and growing independence but also by higher sensitivity to adults' expectations, school performance and competition with peers (46). Adaptation to such stressors requires resilience, which depends on the presence of a healthy group of peers and personal coping strategies. However, when these are lacking, the resulting sense of inadequacy can predispose to somatic symptom disorders (SSDs) or psychiatric comorbidities (i.e., anxiety and depression), which can arise with a subjective sense of fatigue (47, 10). Therefore, SSD and other psychological conditions should be kept high in the list of differential diagnoses of adolescents or older children complaining of fatigue. On the other hand, organic causes must be considered the most prevalent in pre-scholar patients (48).

### Differentiate the types of fatigue through key questions

Different conditions with specific characteristics and definitions may be confused with or described as “fatigue”: muscle weakness, easy fatigability, exercise intolerance and excessive daytime sleepiness (Table S4, Supplementary file S2). They should be addressed and distinguished through specific questions. Finally, the psychological domain should be explored. We, thus, propose some key queries to ask the child complaining of fatigue as part of the diagnostic workflow (Figure 1).

Firstly, the presence of muscle weakness should be investigated and further characterized through the following question: “*Is there any difficulty climbing the stairs or combing hair?*”. Indeed, the incapability of performing such activities, involving the pelvic and shoulder girdle, is suggestive of proximal muscle weakness and should lead the diagnosis towards myopathies or a Guillain Barré syndrome. A thorough neurological examination should always be performed and focused on detecting proximal or distal hyposthenia, as well as manifestations of the first or second motor-neuron syndrome, such as spasticity or alteration of deep tendon reflexes, respectively with hyper or hyporeflexia (49, 50).

As a second question, we propose: “*Is there progressive exhaustibility during an exercise or in the course of the day?*”. This query highlights easy fatigability, a possible hallmark of myasthenia gravis and polymyositis.

In third place, exercise intolerance should be investigated through questions like: “*Is there any difficulty attending gym class or doing physical exercise?*” and “*Do you need to stop soon after having started running or playing a game?*” or “*Do you usually vomit after playing games?*”. Exercise intolerance, which can be thus detected, can be due to alteration of cardiovascular, pulmonary and muscular function or to mitochondrial diseases (51, 52).

Moreover, excessive daytime drowsiness should be addressed by a question about sleep habits and quality. Excessive somnolence has different causes based on the patient's age. During the first months of life, it can be a sign of worsening muscle hypotonia or an underlying metabolic disorder (53). During adolescence, daytime sleepiness is often related to poor sleep hygiene and associated with mental health issues (54, 55). On the contrary, younger children with obstructive sleep apnea syndrome, who lack quantity or quality of sleep, usually present with hyperactive behavior and concentration difficulties (56). Therefore, at this age, when daytime drowsiness is present, it should point toward an organic cause of poor sleep. Lastly, uncontrolled allergic rhinitis, asthma and atopic dermatitis are also known to affect sleep patterns, and should thus be considered in the differential diagnosis of a tired child (57–59).

Furthermore, a specific question addressing drugs being taken is imperative. A thorough medication history must be gathered, with particular focus on drugs which are known to cause fatigue and sedation: benzodiazepines, neuroleptics, anti-hypertensives, antiepileptics and anti-histamines.

Finally, it is mandatory to assess the social and psychological domain: “*Do you regularly attend school?*”, “*Did you recently interrupt previously regular after-school activities?*”, “*Do you have a peer group of friends?*”, “*Do you spend many hours alone in your room using electronic devices?*”. Any positive answer should direct clinicians toward psychological domain impairment and functional disorders, especially if the age is consistent and the patient is an adolescent. School absenteeism, the lack of a group of peers and excessive time spent on electronic media are some of the predominantly recognized markers of children's mental health-related problems, including depression and SSD (60–63). In addition, physical inactivity, sleep alterations and somatic symptoms have been found to predict the development of severe and persistent fatigue in children, further confirming a significant overlap between chronic fatigue and somatic symptom disorders (64). Remarkably, physicians should be aware that SSD can frequently develop in children with a pre-existing chronic illness; when confirmed, it should be addressed and treated through implementation of specific psycho-social measures.

### Consider history and accompanying clinical signs

Fever, night sweats, itching, vomit, diarrhea, weight loss, growth impairment, musculoskeletal pain, joint limitation, or pain can help orient the diagnosis towards a more focused gastrointestinal, oncologic or rheumatological disease field. Worsening school performance may be among the first signs of impaired thyroid function. History elements, clinical signs and red flags to search for in order to rule out the main severe underlying etiologies of fatigue are summarized in Table 1. In their absence, the pediatrician could tend towards a “watchful waiting” approach, with re-evaluation of the child over time.

**Table 1:** **Red flags to search for in a child complaining of fatigue**. Abbreviations: IBD, intestinal bowel disease; SSD, somatic symptom disorder; SMA, spinal muscular atrophy.

Medical history and symptoms	Clinical signs	Diseases
**Abdominal pain, vomiting, diarrhea**, constipation and weight loss; **failure to thrive; polyuria**	Perianal skin tags, gangrenous pyoderma, nodose erythema; oral ulcers, teeth lesions, Duhring dermatitis; bronze skin color, orthostatic hypotension	IBD; celiac disease; Addison's disease
**Bone pain**, limping, **weight loss**, night sweats	**Hepatosplenomegaly**, pallor, **lymphadenopathy**, bruising	Lymphoma, leukemia, tumors
Ophthalmoplegia, impaired vision, **vomit after physical effort**	Retinopathy, exercise intolerance; hepatomegaly	Mitochondrial disease
**Shortness of breath** in toddler; uncertain gait, malaise, arthralgia	Cardiomegaly in toddler, rickety rosary; valgus or varum knee; skin and mucosal petechiae, articular swelling, gum hypertrophy	Rickets; scurvy
**Polyuria, polydipsia**, polypnea, weight loss	**Candida infection** over 1 year of age	Diabetes mellitus; tubulopathy due to metabolic or mitochondrial disease
Weight gain, abdominal pain, **dyspnea after effort**	Hepatomegaly, tachycardia, bradycardia, **peripheral oedemas**	Heart disease
Asthenia or hyposthenia, difficulties in doing stairs or combing hair, uncertain gait, **frequent falls**	**Gottron papules**, heliotropic rash; **Gowers sign**, tendon retraction, **calves hypertrophy**; muscle hypotrophy or hyposthenia; loss of tendon reflexes, hypotonia, muscle weakness	Dermatomyositis; muscle dystrophies; Guillain Barré syndrome; SMA 2-3-4
Pruritus, history of atopy, frequent cutaneous infections	Eczema sparing axillary and groin areas, lichenification, xerosis, Dennie-Morgan folds	Atopic dermatitis
Asthenia, malaise, arthralgia	**Absence of peripheral pulses**	Takayasu's arteritis
**Palpebral ptosis**; constipation, infant age	**Muscle weakness** and **exhaustion; fatigability**; hypotonia	Myasthenia gravis; botulinum toxin intoxication
**School absenteeism**, isolation from peers, history of repeated examinations and medical evaluations	Unremarkable physical examination	SSD; functional disorder; Munchausen syndrome

### Perform a panel of first level blood tests

Figure 1 shows the panel of first-level blood tests that should always be performed on the child complaining of fatigue. When the first-level tests show any alteration, or if they are unremarkable but signs and symptoms are consistent with a specific clinical diagnosis, physicians should go ahead in the diagnostic evaluation, performing second-level tests (Table S5, see Supplementary file S2). However, astute clinicians should always use them appropriately and judiciously, in order to avoid over-medicalization. Remarkably, literature on both adult and pediatric patients advises against performing serologies for cytomegalovirus (CMV), Epstein-Barr virus (EBV) and Lyme disease because hardly any finding could affect the clinical behavior, and results could be confounding (65). On the other hand, in front of suggestive associated symptoms or if the patient is from a place with high endemicity, QuantiFERON or Mantoux test to exclude tuberculosis should be performed.

### Dealing with chronic fatigue syndrome

Once all the possible organic causes at the base of fatigue have been excluded, the literature suggests considering the diagnosis of Chronic Fatigue Syndrome (CFS), a condition also known as myalgic encephalomyelitis, with uncertain etiology and a reported prevalence of 1%–2% in children and adolescents (11). The pediatric diagnostic criteria are less well-defined than adults' ones. Guidelines vary slightly among different groups (24, 25, 66, 67). However, they mostly agree on some key differences compared to the adult guidelines. In particular, the diagnosis of pediatric CFS should be considered when symptoms persist for more than three months (compared to six in the adult population), symptom onset must be gradual, and a debilitating and chronic sense of fatigue should be associated with post-exertional malaise, pain, dizziness, alterations in sleep and decreased neurocognitive performance. Furthermore, all authors include elements to distinguish CFS from “school phobia” (27). Indeed, one of the main issues plaguing children diagnosed with CFS is school absenteeism (68), associated with a severe decrease in general functioning, with some patients even becoming bed-ridden or wheelchair-bound (69). These features suggest an overlap between CFS, SSD, and psychiatric populations. One-third of adolescents diagnosed with CFS are actually affected by mental health problems (70), presenting higher rates of emotional distress, “internalizing” symptoms (mainly anxiety and depression) and personality disorders (71). This overlap is further supported by the fact that SSDs and CFS are more common in female adolescents than males (24). A somatic or psychological rather than organic origin of CFS appears to be likely, considering that the most effective therapeutic intervention was found to be cognitive behavioral therapy (72).

From a pragmatic perspective, we suggest that CFS should be an exclusion diagnosis well before the three months needed to formalize it. Notably, strong consideration should be given to the presence of an underlying SSD, a condition much more prevalent than CFS in the pediatric population (10%–15%) (73–75). A recognized fact is that doctors are often uncomfortable diagnosing SSDs; hence patients with functional disorders are frequently misdiagnosed as having some medically unexplained syndrome (76) or some blurred clinical entity that can be identified on a case-by-case basis within CFS, fibromyalgia, or chronic Lyme disease (77–79). This issue becomes relevant in pediatrics for the associated risks of perpetuating over-medicalization (80) and over-diagnosing the above-cited conditions (81).

## Conclusions

This paper suggests a pragmatic approach to fatigue in children based on age assessment, targeted questions, physical examination clues, and a few laboratory first-level tests. The main limit of this work is that such a clinical algorithm has not been validated on a large population. However, we tried to provide a perspective on the topic offering a succinct and practical method to address a very broad issue. Further studies are needed to investigate the predictive value, specificity and sensitivity of specific diagnostic workflows in managing the child with fatigue.

## Data Availability

The original contributions presented in the study are included in the article/**Supplementary Material**, further inquiries can be directed to the corresponding author/s.
