# Supplementary material for: Approach to the child with fatigue: A focus for the general pediatrician
Source: Front Pediatr. 2022 Dec 2;10:1044170. doi: 10.3389/fped.2022.1044170 (PMC9755349; doi:10.3389/fped.2022.1044170)
Supplement: Supplementary file 1 [file Datasheet1.pdf]

Supplementary file 1.

Table S1. Search strategy and main literature results considered in the perspective.

Database Searched	Search Terms	Exclusion criteria	Main research topic	N° of papers	Concerning adults	Concerning pediatric patients and adolescents	Study type
MEDLINE, Up To Date Google Scholar	(“Fatigue” OR “weakness” OR “tiredness” OR “weariness” OR “drowsiness”) AND (“child” OR “pediatric” OR “paediatric” OR “children” OR “kid” OR “toddler”). “Tired child”; “child with fatigue”; “paediatric fatigue”; “pediatric fatigability”; (“chronic fatigue” AND “children”); (“medication” OR “drugs” AND “fatigue” AND “children”); (“sleep disturbances” AND “children”)	- Articles addressing fatigue related to COVID-19 pandemic<br>- Fatigue in patients with chronic diseases<br>- Duplicates<br>- Case reports, case series	Chronic Fatigue Syndrome/ Myalgic Encephalomyelitis (CSF/ME)	26	4	22	10 review articles, 1 systematic review, 4 guidelines from metanalysis, 11 observational studies
			Fatigue as a clue of psycological/psychiatric problem or Somatic Symptom Disorder	20	6	14	1 systematic review, 5 review articles, 5 commentaries, 9 observational studies
			Fatigue as the main complaint expression of several subtended condition	19	12	7	13 review articles, 3 systematic reviews and 3 observational studies
			**Fatigue as the main complaint ‘per se’**	12	6<br><br>- Approach to the adult patient with fatigue. (UpToDate)<br>- Approach to the patient with muscle weakness (UpToDate)<br>- Approach to the patient with excessive daytime sleepiness (Uptodate)<br>- Finsterer et al. (2004)<br>- Nijrolder et al. (2009)<br>- Sharpe (2002)	6*<br><br>Farmer et al. (2004)<br>Haines et al. (2005)<br>ter Wolbeek et al. (2006)<br>Saidi et al. (2006)<br>Fisher et al. 2013<br>Càrdenas Villamil et al. (2017)	7 clinical review articles, 5 observational studies

*Such literature results are reported in table 3.

Table S2. Literature results concerning fatigue as the main complaint among children and young adults.

Abbreviations: CSF, chronic fatigue syndrome; GP, general practitioner; CSF/ME, chronic fatigue syndrome/myalgic encephalomyelitis; ESR/CRP, erythrocyte sedimentation rate, C- reactive protein; EBV, Epstein Barr virus; ANA, anti-nuclear antibodies; RF, rheumatoid factor; CI, confidence interval; MRC, Medical Research Council.

Citation (Author/Date)	Framework	Research hypotheses	Study type - Methods	Results	Conclusions
Farmer et al. (2004)	Epidemiology of chronic fatiguing illnesses in young people	To estimate the lifetime prevalence of different definitions of chronic fatigue in 8- to17-year-olds.	Observational study. Participants came from two population-based twin series. Parents completed self-report questionnaires that enquired whether either child had ever experienced more than a few days of disabling fatigue. Telephone interviews were undertaken for individuals who had experienced such an episode.	Questionnaires were returned by 1468 families (65% response rate) and telephone interviews were undertaken regarding 99 of the 129 subjects (77%) who had experienced fatigue. The lifetime prevalence estimates ranged from 2.34% (95% CI1.75^2.94) for disabling fatigue lasting 3 months to 1.29% (95% CI 0.87^1.71) for a disorder resembling adult operationally defined chronic fatigue syndrome.	From the age of 11 years, young people have similar rates and types of chronic fatiguing illnesses to adults.<br><br>Chronic fatigue causes considerable impairment in young people, to both social development and time missed from school.<br><br>Operational criteria for chronic fatigue syndrome (CFS) should require only 3 months' symptom duration in young people and not 6 months as in adults.
Haines et al. (2005)	Prevalence of severe fatigue in primary care pediatric patients	To report cases of severe disabling fatigue of over three months in 5–19 year old in primary care and the diagnoses given by the GP	Retrospective cross-sectional study. A postal survey of 1024 UK General Practitioner (GP) practices was undertaken. The number of subjects with disabling fatigue for at least three months with a premorbid level of activity significantly reduced or impaired was reported.	These cases were grouped as: **fatigue diagnoses** (n = 281; 62%) (chronic fatigue, CFS, fatigue/severe fatigue syndrome, fatigue, lethargy, malaise, ME, CFS/ME, fibromyalgic CFS, and fibromyalgia); **viral diagnoses** (viral infection, post-viral fatigue) (n = 154; 34%); and **psychosocial diagnoses** depression, bullying, family factors) (n = 20; 4%).	The prevalence of medically unexplained severe fatigue over three months in 5–19 year old was 62/100 000. Cases were predominantly adolescent girls and were more likely to come from practices in less deprived areas, which could reflect consulting behaviors.

ter Wolbeek et al. (2006)	Prevalence of severe fatigue in adolescents	To determine the prevalence of severe fatigue in adolescents; to explore the role of lifestyle factors in fatigue; to investigate whether severe fatigue in a healthy population is associated with depression, anxiety, and comorbid factors also observed in chronic fatigue syndrome patients.	Observational study. In a sample of 1718 boys and 1749 girls, fatigue severity and duration were measured using a multidimensional questionnaire (Checklist Individual Strength). In addition, self-reports of depressive symptoms, anxiety, chronic fatigue syndrome-related symptoms, and lifestyle characteristics were assessed. Prevalence rates of severe fatigue and severe fatigue for >1 month, based on a clinical cutoff score of the Checklist Individual Strength, were determined for boys and girls separately, and gender-specific predictors of fatigue were identified by multiple regression analysis.	Data showed high prevalence rates of severe fatigue in adolescents. 20.5% of girls and 6.5% of the boys scored above the clinical cutoff score on the Checklist Individual Strength. Of the examined lifestyle characteristics, only sleep characteristics and the participation in sports played a role in predicting fatigue in both genders. Moreover, in girls, fatigue was associated with higher age, an early menarche, medication use, and the absence of an additional job. Overall, girls scored higher on depression, anxiety, and chronic fatigue syndrome-related symptoms. In both girls and boys, the duration of fatigue was positively related to fatigue severity, severity of depression and anxiety, and the number of chronic fatigue syndrome-related symptoms.	Adolescent girls seem to be more vulnerable to symptoms of fatigue and comorbidity than boys. Interestingly, despite a female predominance in complaints, the relation between fatigue and depression, anxiety, and chronic fatigue syndrome-related symptoms was not gender specific and emerged as a cluster. It has been hypothesize that enduring severe fatigue may form a risk factor for the development of chronic fatigue syndrome.
Saidi et al. (2006)	Management of chronic fatigue syndrome in primary care among pediatric patients	To describe characteristics of patients aged 5–19 years with CFS-like illness in primary care and to examine how GPs investigate and manage patients.	Descriptive retrospective study. 62 UK GP practices in the MRC General Practice Research Framework (GPRF) were included. 116 patients consulting a GP with severe fatigue lasting over 3 months were identified. Practice nurses and GPs completed questionnaires from medical notes and patients completed postal questionnaires.	94 patients were considered to meet the Oxford CFS criteria with a fatigue duration of 3 months. 73% were girls, 94% white, mean age was 12.9 years and median illness duration 3.3 years. GPs had principal responsibility for 62%. A diagnosis of CFS/ME was made in 55%, 30% of these within 6 months. Pediatric referrals were made in 82% and psychiatric referrals in 46% (median time of 2 and 13 months respectively). Advice given included setting activity goals, pacing, rest and graded exercise.	Patient characteristics are comparable to those reported in tertiary care, although fewer are severe cases. GPs have responsibility for the majority of patients, are diagnosing CFS/ME within a short time and applying a range of referral and advice strategies. The most ordered lab tests by the GP included: Hemoglobin, White cell count, thyroid, kidney and liver function, , Monospot, ESR/CRP, Urine test, Bone profile, Blood glucose, Immunoglobulins, Creatine kinase.

Fisher. (2013)	Fatigue in pediatrics can be a complaint of an underlying medical condition, psychological or psychiatric condition or a specific sleep disorder.	To provide a framework for evaluating the complaint of fatigue in adolescents offering approaches to management.	Mini-review. It is a communication based on author's personal experience in the clinical management of fatigue in adolescents. They consider: adolescent sleep patterns, psychological and medical causes of fatigue.	A thorough list of possible organic causes of fatigue is considered, along with sleep disorders, psychological causes and medications. Also they underline the need of performing a basic laboratory screening including complete blood count and metabolic panel, as well as thyroid function test. Other possible tests to perform are ESR, EBV antibodies, RF, ANA, pulmonary function tests, chest X-ray or tuberculin test if signs are consistent. Chronic Fatigue syndrome as an exclusion diagnosis is also mentioned.	The evaluation of CFS in adolescents, which applies to the evaluation of fatigue in teenagers in general, incorporates a physical examination that most often demonstrates no abnormalities, as well as basic laboratory tests, which likewise usually demonstrate no abnormalities. In those rare instances in which either the examination or laboratory testing do reveal abnormalities, they may be the clue to a specific diagnosis.
Cárdenas Villamil et al. (2017)	Evaluation and management of the tired adolescent in the family practice	To provide a tool for approaching the adolescent complaining of fatigue.	Narrative review. A total of 33 articles obtained for the diagnostic and therapeutic approach of the tired adolescent in Primary Care were analyzed .	Prevalence of fatigue in adolescence vary from 0.7 to 7.4%. The main cause of fatigue in adolescence was excessive daytime sleepiness and drowsiness for insufficient or inadequate sleep. Other causes to investigate are mentioned.	A sleep assessment should be a routine component of the clinical evaluation of adolescents and the initial approach to exclude secondary causes.

frontiers

List of the main papers evaluated but not all included in the reference list:

CHRONIC FATIGUE SYNDROME/MYALGIC ENCEPHALOMYELITIS (26)

- Avellaneda Fernández A, Pérez Martín A, Izquierdo Martínez M, Arruti Bustillo M, Barbado Hernández FJ, de la Cruz Labrado J, et al. (2009). Chronic fatigue syndrome: aetiology, diagnosis and treatment. BMC Psychiatry, **9** (suppl 1):S1. doi:10.1186/1471-244X-9-S1-S1.
- Bell DS, Jordan K, Robinson M (2001). Thirteen-year follow-up of children and adolescents with chronic fatigue syndrome. *Pediatrics* **107(5)**:994–8. DOI: 10.1542/peds.107.5.994.
- Brigden A, Loades M, Abbott A, Bond-Kendall J, Crawley E (2017). Practical management of chronic fatigue syndrome or myalgic encephalomyelitis in childhood. *Arch Dis Child* **102(10):981-986**. doi:10.1136/archdischild-2016-310622.
- Capelli E, Zola R, Lorusso L, Venturini L, Sardi F, Ricevuti G. Chronic Fatigue Syndrome/Myalgic Encephalomyelitis: An Update (2010). *Int J Immunopathol Pharmacol* **23(4)**:981–9. DOI: 10.1177/039463201002300402.
- Carruthers BM, van de Sande MI, De Meirleir KL, Klimas NG, Broderick G, Mitchell T, et al. (2011). Myalgic encephalomyelitis: International Consensus Criteria. *J Intern Med* **270(4)**:327–38. DOI: 10.1111/j.1365-2796.2011.02428.x.
- Carter BD, Marshall GS (1995). New Developments: Diagnosis and Management of Chronic Fatigue in Children and Adolescents. *Current Problems in Pediatrics* **25:281-93**. Doi:10.1016/s0045-9380(06)80057-5.
- Committee on the Diagnostic Criteria for Myalgic Encephalomyelitis/Chronic Fatigue Syndrome; Board on the Health of Select Populations; Institute of Medicine. Beyond Myalgic Encephalomyelitis/Chronic Fatigue Syndrome: Redefining an Illness. Washington (DC): National Academies Press (US); 2015 Feb 10. 3, Current Case Definitions and Diagnostic Criteria, Terminology, and Symptom Constructs and Clusters. Available from: https://www.ncbi.nlm.nih.gov/books/NBK284898/ [accessed 2022 June 21].
- Evidence based guideline for the management of CFS/ME (chronic fatigue syndrome/myalgic encephalopathy) in children and young people. London: Royal College of Paediatrics and Child Health; 2004. Available from: https://www.whatdotheyknow.com/request/370385/response/925312/attach/5/Appendix%203%20RCPCH%20CFS.pdf [accessed 2022 June 21].
- Fowler T, Duthie P, Thapar A, Farmer A (2005). The definition of disabling fatigue in children and adolescents. *BMC Family Practice* **6:33**. doi:10.1186/1471-2296-6-33.
- Fukuda K, Straus SE, Hickie I, Sharpe MC, Dobbins JG, Komaroff A (1994). The Chronic Fatigue Syndrome: A Comprehensive Approach to Its Definition and Study. *Ann Int Med* **121(12):**953. DOI: 10.7326/0003-4819-121-12-199412150-00009.
- Garralda E, Rangel L, Levin M, Roberts H, Ukoumunne O (1999). Psychiatric adjustment in adolescents with a history of chronic fatigue syndrome. *J Am Acad Child Adolesc Psychiatry* **38(12)**:1515–21. DOI: 10.1097/00004583-199912000-00012.
- Garralda ME, Rangel L (2002). Annotation: Chronic fatigue syndrome in children and adolescents. *J Child Psychol Psychiatry* **43(2)**:169–76. DOI: 10.1111/1469-7610.00010.
- Jason LA, Brown A, Clyne E, Bartgis L, Evans M, Brown M (2012). Contrasting Case Definitions for Chronic Fatigue Syndrome, Myalgic Encephalomyelitis/Chronic Fatigue Syndrome and Myalgic Encephalomyelitis. *Eval Health Prof* **35(3)**:280–304. DOI: 10.1177/0163278711424281.
- Jordan KM, Landis DA, Downey MC, Osterman SL, Thurm AE, Jason LA (1998). Chronic fatigue syndrome in children and adolescents: a review. *J Adolesc Health Off Publ Soc Adolesc Med* **22(1)**:4–18. DOI: 10.1016/S1054-139X(97)00212-7.

- Knight S, Elders S, Rodda J, Harvey A, Lubitz L, Rowe K, et al. (2019). Epidemiology of paediatric chronic fatigue syndrome in Australia. *Arch Dis Child*; **104:733-738**. doi:10.1136/archdischild-2018-316450.
- Knight SJ, Politis J, Garnham C, Scheinberg A, Tollit MA (2018). School Functioning in Adolescents With Chronic Fatigue Syndrome. *Front Pediatr* **6**:302. DOI: 10.3389/fped.2018.00302.
- Knight SJ, Scheinberg A, Harvey AR (2013). Interventions in pediatric chronic fatigue syndrome/myalgic encephalomyelitis: a systematic review. *J Adolesc Health Off Publ Soc Adolesc Med* **53(2)**:154–65. DOI: 10.1016/j.jadohealth.2013.03.009.
- Lievesley K, Rimes KA, Chalder T (2014). A review of the predisposing, precipitating and perpetuating factors in Chronic Fatigue Syndrome in children and adolescents. Clinical Psychology Review **34:233-248**. doi:10.1016/j.cpr.2014.02.002.
- May M, Emond A, Crawley E. (2010). Phenotypes of chronic fatigue syndrome in children and young people. *Arch Dis Child* **95:245-249**. doi:10.1136/adc.2009.158162.
- National Collaborating Centre for Primary Care (UK). Chronic Fatigue Syndrome/Myalgic Encephalomyelitis (or Encephalopathy): Diagnosis and Management of Chronic Fatigue Syndrome/Myalgic Encephalomyelitis (or Encephalopathy) in Adults and Children [Internet]. London: Royal College of General Practitioners (UK); 2007 [cited 2022 May 20]. (National Institute for Health and Clinical Excellence: Guidance). Available from: http://www.ncbi.nlm.nih.gov/books/NBK53577/ [accessed 2022 June 21].
- NICE (National Institute for Health and Care Excellence) guideline; Royal College of Physicians. Myalgic encephalomyelitis (or encephalopathy)/chronic fatigue syndrome: diagnosis and management (NG206). Published: 29 October 2021. Available from: www.nice.org.uk/guidance/ng206 [accessed 2022 June 21]
- Rangel L, Garralda ME, Levin M, Roberts H (2000). The course of severe chronic fatigue syndrome in childhood. *J R Soc Med* **93(3)**:129–34. DOI: 10.1177/014107680009300306.
- Rollnik JD. Chronic Fatigue Syndrome: A Critical Review (2017). *Fortschr Neurol Psychiatr* **85(2)**:79–85. DOI: 10.1055/s-0042-121259.
- Sankey A, Hill CM, Brown J, Quinn L, Fletcher A (2006). A follow-up study of chronic fatigue syndrome in children and adolescents: symptom persistence and school absenteeism. *Clin Child Psychol Psychiatry* **11(1)**:126–38. DOI: 10.1177/1359104506059133.
- Serafimova T, Loades M, Gaunt D, Crawley E (2021). Who should we ask about mental health symptoms in adolescents with CFS/ME? Parent-child agreement on the revised children's anxiety and depression scale. *Clin Child Psychol Psychiatry* **26(2)**:367–80. DOI: 10.1177/1359104521994880.
- Wilson A, Hickie I, Lloyd A, Hadzi-Pavlovic D, Boughton C, Dwyer J, et al. (1994). Longitudinal study of outcome of chronic fatigue syndrome. *BMJ* **308(6931)**:756–9. DOI: 10.1136/bmj.308.6931.756.

FATIGUE AS THE MAIN ISOLATED COMPLAINT (12)

- Cárdenas Villamil JP, Cavanzo Ramírez AI, García Manrique JG (2017). El adolescente "cansado": evaluación y manejo en al consulta de medicina familiar. *Semergen*. **1138-3593**. http://dx.doi.org/10.1016/j.semerg.2017.01.009
- Chervin RD. Approach to the patient with excessive daytime sleepiness. In: *UpToDate*, Scammell TE, Eichler AF (Ed), UpToDate, Waltham, MA, 2022.
- Farmer A, Fowler T, Scourfield J, Thapar A (2004). Prevalence of chronic disabling fatigue in children and adolescents. *Br J Psychiatry* **184(6):477–81**. DOI: 10.1192/bjp.184.6.477.
- Finsterer J, Mahjoub SZ (2014). Fatigue in Healthy and Diseased Individuals. *Am J Hosp Palliat Med* **31(5)**:562–75. DOI: 10.1177/1049909113494748.

- Fisher M (2013). Fatigue in adolescents. Mini-review. *J Pediatr Adolesc Gynecol* **26 (252-256)**. doi:10.1016/j.jpag.2011.12.067.
- Fosnocht KM, Ende J. Approach to the adult patient with fatigue. In: *UpToDate*, Elmore JG and Givens J (Ed), UpToDate, Waltham, MA, 2022.
- Haines LC (2005). Prevalence of severe fatigue in primary care. *Arch Dis Child* **90(4)**:367–8. DOI: 10.1136/adc.2003.039917.
- Nijrolder I, van der Windt D, de Vries H, van der Horst H (2009). Diagnoses during follow-up patients presenting with fatigue in primary care. *CMAJ*, **181(10)**. DOI: 10.1503/cmaj.090647.
- Saidi L and Haines L (2006). The management of children with chronic fatigue syndrome-like illness in primary care: a cross-sectional study. *British Journal of General Practice*; **56:43-47.** PMID: 16438814.
- Sharpe M, Wilks D. Fatigue (2002). *BMJ*, **325(7362)**:480–3. DOI: 10.1136/bmj.325.7362.480.

FATIGUE AS EXPRESSION OF OTHER SUBTENDED CONDITIONS - NEUROLOGIC FATIGUE AND PATHOPHYSIOLOGY OF FATIGUE (19)

- Barohn R, Dimachkie M, Jackson C (2014). A Pattern Recognition Approach to Patients with a Suspected Myopathy. *Neurologic Clinics* **32(3):**569–593. DOI: 10.1016/j.ncl.2014.04.008.
- Chaudhuri A, Behan PO (2004). Fatigue in neurological disorders. *Lancet*; **363:978-88.** Doi:10.1016/S0140-6736(04)15794-2.
- Davis MP, Walsh D (2010). Mechanisms of fatigue. *J Support Oncol* 8(4):164–74. PMID: 20822034.
- Janssens L, Brumagne S, McConnell AK, Raymaekers J, Goossenns N, Gayan-Ramirez G, et al. (2013). The assessment of inspiratory muscle fatigue in healthy individuals: A systematic review. *Respir Med* **107(3)**:331–46. DOI: 10.1016/j.rmed.2012.11.019.
- Jason LA, Evans M, Brown M, Porter N (2010). What is Fatigue? Pathological and Nonpathological Fatigue. *M&R* **2(5)**:327–31. DOI: 10.1016/j.pmrj.2010.03.028.
- Kluger BM, Krupp LB, Enoka RM (2013). Fatigue and fatigability in neurologic illnesses: Proposal for a unified taxonomy. *Neurology* **80(4)**:409–16. DOI: 10.1212/WNL.0b013e31827f07be.
- Mancuso M, Angelini C, Bertini E, Carelli V, Comi GP, Minetti C, et al. (2012). Fatigue and exercise intolerance in mitochondrial diseases. Literature revision and experience of the Italian Network of mitochondrial diseases. *Neuromuscular Disorders* **22**:S226–S229. DOI: 10.1016/j.nmd.2012.10.012.
- Meltzer LJ, Pugliese CE (2017). Sleep in young children with asthma and their parents. *J Child Health Care*. **21(3):**301-311. DOI:10.1177/1367493517712064.
- Murray BJ. Excessive daytime sleepiness due to medical disorders and medications. In: *UpToDate*, Scammel TE, Eichler AF (Ed), UpToDate, Waltham, MA, 2022.
- Owens J, Adolescent Sleep Working Group, Committee on Adolescence (2014). Insufficient Sleep in Adolescents and Young Adults: An Update on Causes and Consequences. *Pediatrics* **134(3)**:e921–32. DOI: 10.1542/peds.2014-1696.
- Owens S, Gutin B (2000). Exercise Intolerance. *Pediatrics in Review* **21(1)**:6–9. DOI: 10.1542/pir.21-1-6.
- Penner IK, Paul F (2007). Fatigue as a symptom or comorbidity of neurological diseases. *Nat Rev Neurol* **13(11)**:662–75. DOI: 10.1038/nrneurol.2017.117.
- Ramirez FD, Chen S, Langan SM, Prather AA, McCulloch CE, Kidd SA, et al (2019). Association of Atopic Dermatitis With Sleep Quality in Children. *JAMA Pediatr*. **173(5):**e190025. DOI:10.1001/jamapediatrics.2019.0025.
- Shefner JM. Approach to the patient with muscle weakness. In: *UpToDate*, Targoff IN and Wilterdink JL (Ed), UpToDate, Waltham, MA, 2022.

- Siniscalchi A, Gallelli L, Russo E, De Sarro G (2013). A review on antiepileptic drugs-dependent fatigue: Pathophysiological mechanisms and incidence. *Eur J Pharmacol* **718(1-3):**10–6. DOI: 10.1016/j.ejphar.2013.09.013.
- Takahashi T, Yamada K, Kobayashi H, Hasegawa Y, Taketani T, Fukuda S, et al. (2015). Metabolic disease in 10 patients with sudden unexpected death in infancy or acute life-threatening events: SUDI and ALTE in metabolic disease. *Pediatr Int,* **57(3)**:348–53. DOI: 10.1111/ped.12660.
- Tanaka M, Watanabe Y. Supraspinal regulation of physical fatigue (2012). *Neurosci Biobehav Rev* **36(1)**:727–34. DOI: 10.1016/j.neubiorev.2011.10.004.
- Teoh H, Carey K, Sampaio H, Mowat D, Roscioli T, Farrar M (2017). Inherited Paediatric Motor Neuron Disorders: Beyond Spinal Muscular Atrophy. *Neural Plasticity* **2017**:6509493. DOI: 10.1155/2017/6509493.
- ter Wolbeek M, van Doornen LJP, Kavelaars A, Heijnen CJ (2006). Severe fatigue in adolescents: a common phenomenon? *Pediatrics,* **117(6)**:e1078-1086. DOI: 10.1542/peds.2005-2575.
- Urbano G, Tablizo B, Moufarrej Y, Tablizo M, Chen M, Witmans M (2021). The Link between Pediatric Obstructive Sleep Apnea (OSA) and Attention Deficit Hyperactivity Disorder (ADHD) *Children* **8(9)**:824. DOI: 10.3390/children8090824.

FATIGUE AS A CLUE OF PSYCHIATRIC/PSYCHOLOGICAL/SOMATIC DISORDERS (20)

- Bakker RJ, van de Putte EM, Kuis W, Sinnema G (2009). Risk factors for persistent fatigue with significant school absence in children and adolescents. *Pediatrics* **124(1)**:e89–95. DOI: 10.1542/peds.2008-1260.
- Caqueo-Urízar A, Flores J, Escobar C, Urzúa A, Irarrázaval M (2020). Psychiatric disorders in children and adolescents in a middle-income Latin American country. *BMC Psychiatry* **20(1)**:104. DOI: 10.1186/s12888-020-02512-4.
- Cozzi G, Barbi E (2020). Chronic school absenteeism as a diagnostic clue for paediatricians. *J Paediatr Child Health* **56(2)**:191–3. DOI: 10.1111/jpc.14689.
- Cozzi G, Minute M, Skabar A, Pirrone A, Mohamad J, Neri E, et al. (2017). Somatic symptom disorder was common in children and adolescents attending an emergency department complaining of pain. *Acta Paediatr* **106(4)**:586–93. DOI: 10.1111/apa.13741.
- De Sanctis V, Abbasciano V, Soliman AT, Soliman AT, Soliman N, Di Maio S, et al. (2019). The juvenile fibromyalgia syndrome (JFMS): a poorly defined disorder. A*cta Bio Medica Atenei Parm* **90(1)**:134–48. DOI: 10.23750/abm.v90i1.8141.
- Joyner MJ (2016). Fatigue: Where did we come from and how did we get here? Medicine & Science in Sports & Exercise. doi: 10.1249/MSS.0000000000000938.
- Jureidini JN, Shafer AT, Donald TG (2003). "Munchausen by proxy syndrome": not only pathological parenting but also problematic doctoring? *Med J Aust*;**178(3)**:130–2. DOI: 10.5694/j.1326-5377.2003.tb05104.x.
- Kleppang AL, Steigen AM, Ma L, Søberg Finbråten H, Hagquist C (2021). Electronic media use and symptoms of depression among adolescents in Norway. *PLOS ONE* **16(7)**:e0254197. DOI: 10.1371/journal.pone.0254197.
- Lemola S, Perkinson-Gloor N, Brand S, Dewald-Kaufmann JF, Grob A (2015). Adolescents' Electronic Media Use at Night, Sleep Disturbance, and Depressive Symptoms in the Smartphone Age. *J Youth Adolesc* **44(2)**:405–18. DOI: 10.1007/s10964-014-0176-x.
- Liao SC, Ma HM, Lin YL, Huang WL (2019). Functioning and quality of life in patients with somatic symptom disorder: The association with comorbid depression. *Compr Psychiatry* **90**:88–94. DOI: 10.1016/j.comppsych.2019.02.004.
- Malas N, Ortiz-Aguayo R, Giles L, Ibeziako P (2017). Pediatric Somatic Symptom Disorders. *Curr Psychiatry Rep* **19(2)**:11. DOI: 10.1007/s11920-017-0760-3.

- Morabito G, Barbi E, Cozzi G (2020). The Unaware Physician's Role in Perpetuating Somatic Symptom Disorder. *JAMA Pediatr* **174(1)**:9. DOI: 10.1001/jamapediatrics.2019.4381.
- Murray AM, Toussaint A, Althaus A, Löwe B (2013). Barriers to the diagnosis of somatoform disorders in primary care: protocol for a systematic review of the current status. *Syst Rev*;**2**:99. DOI: 10.1186/2046-4053-2-99.
- Murray AM, Toussaint A, Althaus A, Löwe B (2016). The challenge of diagnosing non-specific, functional, and somatoform disorders: a systematic review of barriers to diagnosis in primary care. Journal of Psychosomatic Research. **80:1-10.** Doi:10.1016/j.jpsychores.2015.11.002.
- Peri F, Nisticò D, Morabito G, Occhipinti A, Ventura A, Barbi E, et al. (2019). Somatic symptom disorder should be suspected in children with alleged chronic Lyme disease. *Eur J Pediatr* **178(8)**:1297–300. DOI: 10.1007/s00431-019-03416-6.
- Roach A (2018). Supportive Peer Relationships and Mental Health in Adolescence: An Integrative Review. *Issues Ment Health Nurs* **39(9)**:723–37. DOI: 10.1080/01612840.2018.1496498.
- Silber TJ, Pao M (2003). Somatization Disorders in Children and Adolescents. *Pediatr Rev* 2**4(8)**:255–64. DOI: 10.1542/pir.24-8-255.
- Taastrøm A, Klahn J, Staal N, Thomsen PH, Johansen A (2014). Children and adolescents in the Psychiatric Emergency Department: a 10-year survey in Copenhagen County. *Nord J Psychiatry* **68(6)**:385–90. DOI: 10.3109/08039488.2013.846410.
- Tack M (2019). Medically Unexplained Symptoms (MUS): Faults and Implications. *Int J Environ Res Public Health* **16(7)**:1247. DOI: 10.3390/ijerph16071247.
- Taylor RR, Jason LA, Jahn SC (2003). Chronic fatigue and sociodemographic characteristics as predictors of psychiatric disorders in a community-based sample. *Psychosom Med* **65(5)**:896–901. DOI: 10.1097/01.psy.0000088580.28749.7f.

OTHERS:

- Related to "FATIGUE IN PATIENTS WITH SPECIFIC CONDITIONS"

- Crichton A, Knight S, Oakley E, Babl FE, Anderson V (2015). Fatigue in Child Chronic Health Conditions: A Systematic Review of Assessment Instruments. *Pediatrics* **135(4)**:e1015–31. DOI: 10.1542/peds.2014-2440.
- Dantzer R, Capuron L, Irwin MR, Miller AH, Ollat H, Perry VH, et al. (2008). Identification and treatment of symptoms associated with inflammation in medically ill patients. *Psychoneuroendocrinology* **33(1)**:18–29. DOI: 10.1016/j.psyneuen.2007.10.008.
- Gagner C, Landry-Roy C, Lainè F and Beauchamp MH (2015). Sleep-Wake Disturbances and Fatigue after Pediatric Traumatic Brain Injury: A Systematic Review of the Literature. *Journal of Neurotrauma* **32:1-14**. DOI: 10.1089/neu.2014.3753.
- Houghton KM, Tucker LB, Potts JE, McKenzie DC (2008). Fitness, fatigue, disease activity, and quality of life in pediatric lupus. *Arthritis Rheum* **59(4)**:537–45. DOI: 10.1002/art.23534.
- Molloy MA, DeWitt ES, Morell E, Reichman JR, Brown DW, Kobayashi R, et al. (2021). Parent-reported symptoms and perceived effectiveness of treatment in children hospitalized with advanced heart disease. *J Pediatr*; **238:221-7**. doi:10.1016/j.jpeds.2021.06.077.
- Nap-van der Vlist MM, Burghard M, Hulzebos HJ, Doeleman WR, Heijerman HGM, van der Ent CK, et al. (2018). Prevalence of severe fatigue among adults with cystic fibrosis: A single center study. *J Cyst Fibros* **17(3)**:368–74. DOI: 10.1016/j.jcf.2018.03.003.

- Nap-van der Vlist MM, Dalmeijer GW, Grootenhuis MA, van der Ent CK, van den Heuvel-Eibrink MM, Wulffraat NM, et al. (2019). Fatigue in childhood chronic disease. *Arch Dis Child* **104(11)**:1090–5. DOI: 10.1136/archdischild-2019-316782.
- Nap-van der Vlist MM, Dalmeijer GW, Grootenhuis MA, van der Ent K, van den Heuvel-Eibrink MM, Swart JF, et al. (2021). Fatigue among children with a chronic disease: a cross-sectional study. *BMJ Paediatrics Open*; **5:e000958**. doi:10.1136/ bmjpo-2020-000958.
- Nijhof LN, van de Putte EM, Wulffraat NM, Nijhof SL (2016). Prevalence of Severe Fatigue Among Adolescents With Pediatric Rheumatic Diseases. *Arthritis Care Res (Hoboken)*, **68(1):**108-114. DOI:10.1002/acr.22710.
- Özdel S, Özçakar ZB, Cakar N, Aydın F, Çelikel E, Elhan AH et al. (2018). Fatigue in Pediatric Patients with Familial Mediterranean Fever. *Modern Rheumatology*. **28(6):** 1016-1020. DOI: 10.1080/14397595.2018.1427459.
- Robinson PD, Oberoi S, Tomlinson D, Duong N, Davis H, Cataudella D, et al. (2018). Management of fatigue in children and adolescents with cancer and in paediatric recipients of haemopoietic stem-cell transplants: a clinical practice guideline. *Lancet Child Adolesc Helath*; **2(5):371-378**. Doi: 10.1016/S2352-4642(18)30059-2.
- Sun R, Liu M, Lu L, Zheng Y, Zhang P (2015). Congenital Heart Disease: Causes, Diagnosis, Symptoms, and Treatments. *Cell Biochem Biophys* **72(3)**:857–60. DOI: 10.1007/s12013-015-0551-6.
- Tarakci E, Arman N, Barut K, Şahin S, Adroviç A, Kasapçopur Ö. (2019) Fatigue and sleep in children and adolescents with juvenile idiopathic arthritis: a cross-sectional study. *Turk J Med Sci* (**49):58-65**. doi:10.3906/sag-1711-167.
- Van de Vijver E, Van Gils A, Beckers L, Van Driessche Y, Moes ND, van Rheenen PF (2019). Fatigue in children and adolescents with inflammatory bowel disease. *World J Gastroenterol* **25(5)**:632–43. doi: 10.3748/wjg.v25.i5.632.
- Van Dijk−Lokkart EM, Steur LMH, Braam KI, Veening MA, Huisman J, Takken T, et al. (2019) Longitudinal development of cancer‐related fatigue and physical activity in childhood cancer patients. *Pediatr Blood Cancer* **66(12):e27949**. DOI: 10.1002/pbc.27949.
- Varni JW, Limbers CA, Bryant WP and Wilson DP (2010). The PedsQL^{TM} Multidimensional Fatigue Scale in pediatric obesity: Feasibility, reliability and validity. *International Journal of Pediatric Obesity* **5:34-42**. Doi: 10.3109/17477160903111706.
- Varni JW, Limbers CA, Bryant WP, Wilson DP (2009). The PedsQL Multidimensional Fatigue Scale in type 1 diabetes: feasibility, reliability, and validity. *Pediatr Diabetes* **10(5)**:321–8. DOI: 10.1111/j.1399-5448.2008.00482.x.
- Vassallo G, Mughal Z, Robinson L, Weisberg D, Roberts SA, Hupton E, et al. (2019). Perceived fatigue in children and young adults with neurofibromatosis type 1. *J Paediatr Child Health* (**56):878-883**. doi:10.1111/jpc.14764.

- Related to “FATIGUE AND COVID19”

- Bignardi G, Dalmaijer ES, Anwyl-Irvine AL, Smith TA, Siugzdaite R, Uh S, et al. (2020). Astle DE. Longitudinal increases in childhood depression symptoms during the COVID-19 lockdown. *Arch Dis Child*;**106(8)**:791–797. DOI: 10.1136/archdischild-2020-320372.
- Brackel CLH, Lap CR, Buddingh EP, van Houten MA, var der Sande LJTM, Langereis EJ, et al. (2021). Pediatric long-COVID: an overlooked phenomenon? Pediatric Pulmonology; **56:2495-2502**. doi:10.1016/S0140-6736(04)15794-2.
