# Supplementary material for: Approach to the child with fatigue: A focus for the general pediatrician
Source: Front Pediatr. 2022 Dec 2;10:1044170. doi: 10.3389/fped.2022.1044170 (PMC9755349; doi:10.3389/fped.2022.1044170)
Supplement: Supplementary file 2 [file Datasheet2.pdf]

Supplementary file 2.

Table S3. Classification of physical fatigue.

Abbreviations: CNS, central nervous system; MS, multiple sclerosis; TBI, traumatic brain injury; SMA, spinal muscular atrophy; CO, carbon monoxide.

**CENTRAL NEUROLOGICAL FATIGUE**	Dysfunction of specific cortical or subcortical networks, due to spinal or supraspinal disfunction. It can overlap with mental fatigue.	MS, Parkinson disease, cerebrovascular ischemia, mitochondrial disorders, hereditary spastic paraplegias, spinocerebellar ataxias, CNS infections and tumors, TBI
**PERIPHERAL NEUROLOGICAL FATIGUE**	Second motor neuron dysfunction, peripheral nerves, neuromuscular junction, or muscular diseases.<br>Keep attention to distinguishing **easy fatigability** from **muscle weakness**. Muscle weakness can be expression of both central and peripheral fatigue (see the text).	SMA, polyradiculopathies, plexopathies, polyneuropathies due to inflammatory process such as vasculitis, myasthenia gravis, Guillan Barrè, toxic exposure (CO), nutritional deficits (B12, copper, zinc), myopathies, or rhabdomyolysis, mitochondrial disorders
**NON-NEUROLOGICAL FATIGUE**	It can be due to pro-inflammatory cytokines, anemia, hormone deficiencies, poor nutrition, vitamin deficiencies, toxins, and poor tissue perfusion. It also includes drug-related fatigue.<br>It can overlap with mental fatigue.	Infectious, hematologic, gastroenterological, rheumatological, and endocrine disorders, malignancies, heart failure, kidney failure.<br>Drugs: benzodiazepines (i.e. clonazepam, clobazam, lorazepam and diazepam), anti-epileptics (i.e. phenobarbital, primidone, vigabatrin, gabapentin and levetiracetam), neuroleptics (i.e. chlorpromazine), anti-hypertensives (i.e. clonidine and labetalol), antihistamine drugs (anti-H1 and anti-H2, i.e. cetirizine).

Table S4. Definition of different connotations of fatigue and more frequently associated conditions.

Abbreviations: CNS, central nervous system; OSAS, obstructive sleep apnea syndrome;

**MENTAL FATIGUE**	Subjective sense of weariness.	Depression, psychiatric comorbidities, neoplastic, endocrinological, metabolic, inflammatory, and other organic conditions
**EASY FATIGABILITY**	Reduced capacity to maintain activity, exhaustion. Muscle strength is restored after rest.	Myasthenia gravis, dermatomyositis, polymyositis
**MUSCLE WEAKNESS**	Lack of or reduced muscle strength	Myopathies, peripheral neuropathies and CNS diseases
**EXERCISE INTOLERANCE**	Incapability of sustaining a physical activity after begun	Mitochondrial, hearth, lung disease, muscle dystrophy
**DAYTIME SOMNOLENCE**	Secondary to sleep disturbances	OSAS, allergic rhinitis, atopic dermatitis, asthma, depression, narcolepsy, addiction

Table S5. The panel of 1st level exams, possible underlying conditions, and 2nd level diagnostic tests.

Abbreviations: MCV, mean corpuscular volume; ESR, erythrocyte sedimentation rate; CRP, C-reactive protein; IBDs, inflammatory bowel diseases; US, ultrasound; MRI, magnetic resonance imaging; CNS, central nervous system; ACTH, adrenocorticotropic hormone; AST, aspartate transaminase; ALT, alanine aminotransferase; CPK, creatine phosphokinase; HBsAg, surface antigen of hepatitis B; HCV, hepatitis C virus, HAV, hepatitis A virus; HEV, hepatitis E virus; EBV, Epstein-Barr virus; ADV, adenovirus, ANA, antinuclear antibodies; SMA, smooth muscle antibodies; LKM1, liver kidney microsome type 1; LC1, liver-cytosol type 1; GGT, gamma glutamyl transferase; LDH, lactate dehydrogenase; TTG, tissue transglutaminase; EMA, endomysial antibody; EGDS, esophagogastroduodenoscopy; TSH, thyroid-stimulating hormone; ALP, alkaline phosphatase; MDs, muscular dystrophies, PCR, polymerase chain reaction; TB, tuberculosis.

FIRST LEVEL LAB TESTS	CLINICAL CONDITION	SECOND LEVEL TESTS
**Complete Blood Count with MCV**	Anemia; leukemia	Blood smear, bone marrow aspirate, B12 and folate
**Serum iron levels and transferrin**	Iron deficiency anemia	Vitamin C levels, oriented tests to exclude malabsorption
**ESR, CRP**	Infective or inflammatory disorders (i.e. IBDs, vasculitis); lymphoma or other neoplasia; obesity (high ESR levels described, first exclude other causes)	Fecal calprotectin and abdomen US (to detect bowel thickness, lymph nodes, masses); chest X-ray (for mediastinum to rule out isolated lymphoma and vasculitis); brain MRI (lymphoma, CNS tumor); echocardiography (vasculitis)
**Fasting glycemia**	Diabetes mellitus or hypoglycemia	Urinary ketones, insulinemia/glycemia ratio
**Electrolytes, creatinine, urine dipstick test**	Renal disease, tubulopathy; Addison's disease	Abdomen US, urinary beta2-microglobulin, albumin, amino acids and glucose levels; morning ACTH and urinary free cortisol
**AST, ALT**	Liver disease (viral, autoimmune, Wilson disease); muscle disease (primarily associated with CPK elevation)	HBsAg, HCV, HAV, HEV, EBV, ADV serology; ANA, SMA, LKM1, LC1 antibodies; ceruloplasmin; GGT and abdomen US (to assess cholestasis); CPK, Aldolase, LDH (especially if AST>ALT)
**TTG IgA and total IgA levels**	Celiac disease	EMA; EGDS
**TSH**	Hypothyroidism or hyperthyroidism	Anti-thyroid peroxidase and anti-thyroglobulin antibodies; thyroid US
**ALP**	Rickets	Long-bone X-ray; echocardiography (heart failure for cardiomyopathy in toddlers)
**CPK**(82)	• Mild elevation (CPK < 600): polymyositis, dermatomyositis<br>• Mild-moderate elevation (CPK < 600 or 600-1500 U/L): facioscapulohumeral dystrophy, myotonic dystrophies, some limb-girdle dystrophies, Pompe disease, paraneoplastic myositis, viral myositis<br>• Severe elevation (CPK ~ 10 000 U/L): Dystrophinopathies (Becker and Duchenne MDs), limb-girdle dystrophies, McArdle disease<br>• Extremely severe elevation (CPK ~ 200 000 U/L): rhabdomyolysis<br><br>For any level of increase, if fatigue is present, always search for muscle weakness, Gower sign, Gottron papules, and heliotropic rash.	Aldolase, AST, ALT, LDH (inflammatory or degenerative myopathies); myoglobinuria (rhabdomyolysis); muscle biopsy; viral PCR and serology: Influenza virus, Coxsackie virus, enterovirus (viral myositis)
**Mantoux test / QuantiFERON TB** – only if endemic geographical area or suggestive symptoms	Tuberculosis	Chest X ray
